# Validation of an instrument prototype for the minimally invasive fetal surgery of gastroschisis in an inanimate model

**DOI:** 10.1007/s00464-025-12100-w

**Published:** 2025-08-19

**Authors:** Andreas Meinzer, Lidya-Olgu Durmaz, Eva Ana Menke, Helene Zerwer, Gerhard Schmidt, Matthias Burmeister, Gennadii Ivanov, Malte Alexander Lang, Celine Viviane Greitens, Thomas Franz Krebs, Ibrahim Alkatout, Robert Bergholz

**Affiliations:** 1 Department of General, Visceral, Thoracic, Transplant and Pediatric Surgery, UKSH University Hospital of Schleswig-Holstein Kiel Campus, Arnold-Heller-Strasse 3, 24105 Kiel, Germany; 2 Kurt Semm Centre for Laparoscopic and Robot Assisted Surgery, UKSH University Hospital of Schleswig-Holstein Kiel Campus, Arnold-Heller-Strasse 3, 24105 Kiel, Germany; 3 Institute of Electrical Engineering and Information Technology, Christian-Albrecht-University of Kiel, Kaiserstrasse 2, 24143 Kiel, Germany; 4 Department of Pediatric Surgery, Ostschweizer Children’s Hospital, Claudiusstrasse 6, 9006 St. Gallen, Switzerland; 5 Department of Gynecology and Obstetrics, UKSH University Hospital of Schleswig-Holstein Kiel Campus, Arnold-Heller-Strasse 3, 24105 Kiel, Germany

**Keywords:** Prenatal surgery, Minimally invasive surgery, Fetoscopic surgery, In utero intervention, Amnioinfusion, Complex gastroschisis

## Abstract

**Introduction:**

Complex gastroschisis requires timely intervention to protect the fetal intestine from inflammation and strangulation and avoid viscero-abdominal disproportion (VAD). Earlier results in ovine models for the fetoscopic management of gastroschisis highlight the benefits of minimally invasive coverage; yet specialized instruments appear to be needed for better procedural execution. The aim of this study was to create and validate a first prototype instrument for the prenatal covering of the protruded intestines in gastroschisis.

**Methods:**

A 7-mm diameter fetoscopic instrument was designed to hold and deploy a protective bag over the gastroschisis defect after suture fixation to the fetus*.* An inanimate model was used to evaluate the instrument’s usability and effectiveness: Eleven participants performed bag placement and suturing both with and without the prototype, enabling a comparative assessment of procedural performance. Statistical analysis was conducted to evaluate the duration of the procedure, while product deficiencies were qualitatively assessed using a Likert-scale questionnaire. The overall usability of the prototype was further evaluated using the system usability scale (SUS).

**Results:**

The prototype consistently enhanced bag handling and positioning. Median procedural time slightly increased from 118.5 to 120.5 s with the prototype (*p* = 0.98), without affecting the overall procedural efficiency. Usability assessments using the SUS (median score: 67.95) and the Likert scale indicated a generally favorable response. Importantly, usability ratings were consistent regardless of participants’ prior experience in minimally invasive surgery (*p* = 0.43), underscoring the intuitive design and ease of adoption of the prototype.

**Conclusion:**

Despite a minor increase in procedural time, the prototype enabled secure bag placement and demonstrated moderate usability across all participants. This is particularly relevant for fetal procedures requiring amnioinfusion, as opposed to partial amniotic carbon dioxide insufflation (PACI) used in the inanimate model. However, further mechanical refinement is warranted to enhance performance and address usability concerns.

Gastroschisis is a congenital anomaly characterized by herniation of fetal intestinal loops through an abdominal wall defect. Its etiology is multifactorial, with maternal factors such as young age, low body mass index, and low socioeconomic status, along with environmental exposures, including infections and pollutants, contributing to risk. However, no genetic or syndromic associations have not yet been identified [1–3].

Approximately, 25% of cases are classified as complicated gastroschisis, defined by the presence of intestinal necrosis, volvulus, atresia, or perforation. These cases are associated with intrauterine growth restriction, neonatal developmental delays, and increased postnatal morbidity and mortality [4]. Intrauterine surgical intervention aims to protect the intestine from amniotic fluid exposure, limit extra-abdominal bowel growth, and reduce viscero-abdominal disproportion (VAD) [5, 6].

Previous experimental studies demonstrated that fetoscopic coverage using natural latex bags—analogous to postnatal secondary closure—can reduce inflammation, increase interstitial cells of Cajal (ICC) density, and promote gradual intrauterine repositioning of herniated bowel [7]. Despite partial bag dislocation near term, VAD was reduced without inducing abdominal compartment syndrome. This approach results in a smaller, non-inflamed intestine and a larger abdominal cavity at birth, facilitating postnatal repair and reducing complex gastroschisis incidence.

However, technical challenges—particularly secure bag fixation in a fluid-filled environment without partial carbon dioxide insufflation (PACI)—have limited reproducibility and clinical translation, necessitating improved instrumentation [8]. Our group previously conceptualized a specialized fetoscopic instrument tailored to enhance bag placement and fixation (Fig. 1) [6, 7].

**Fig. 1:**
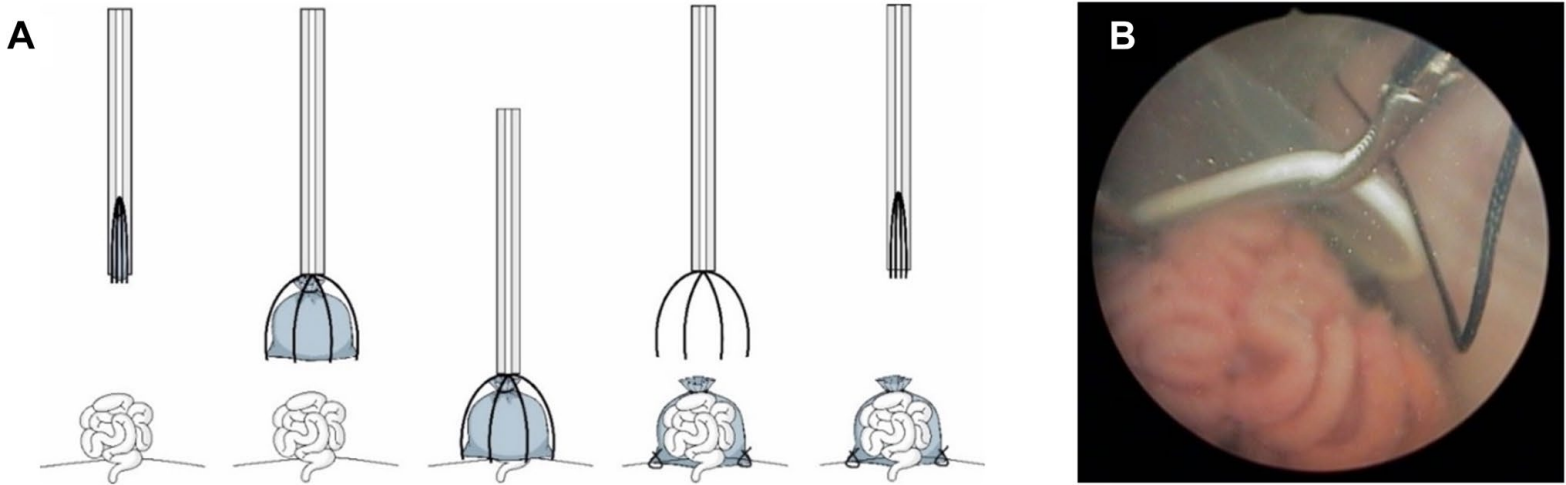
**A** sketch of the instrument for fetoscopic procedures: expandable bag over wires, inserted via port, maneuvered over prolapsed bowel loops, and secured by suturing before retrieval. **B** prenatal covering of gastroschisis in ovine model. **A** and **B** are adapted from Bergholz et al. [4]

This study aims to validate fetoscopic coverage of gastroschisis using an exclusively designed instrument prototype in an inanimate model. Results are expected to inform future technical refinements in prenatal gastroschisis repair and support the development of dedicated application-specific tools.

## Methods

To test the engineered instrument prototype, an inanimate model simulating a fetus with gastroschisis inside a uterus with partial carbon dioxide insufflation (PACI) was developed. This model was based on the low-fidelity fetoscopic surgical simulator previously described by Patel et al. [9].

### Experimental setup

This feasibility study was conducted at the University Hospital of Schleswig–Holstein, Kiel Campus, in cooperation with the Institute of Electrical Engineering and Information Technology, Christian-Albrechts-University of Kiel. As no animals or human subjects were involved, the local ethics committee confirmed that no formal ethical approval or institutional review board clearance was required.

### The instrument prototype

**Fig. 2:**
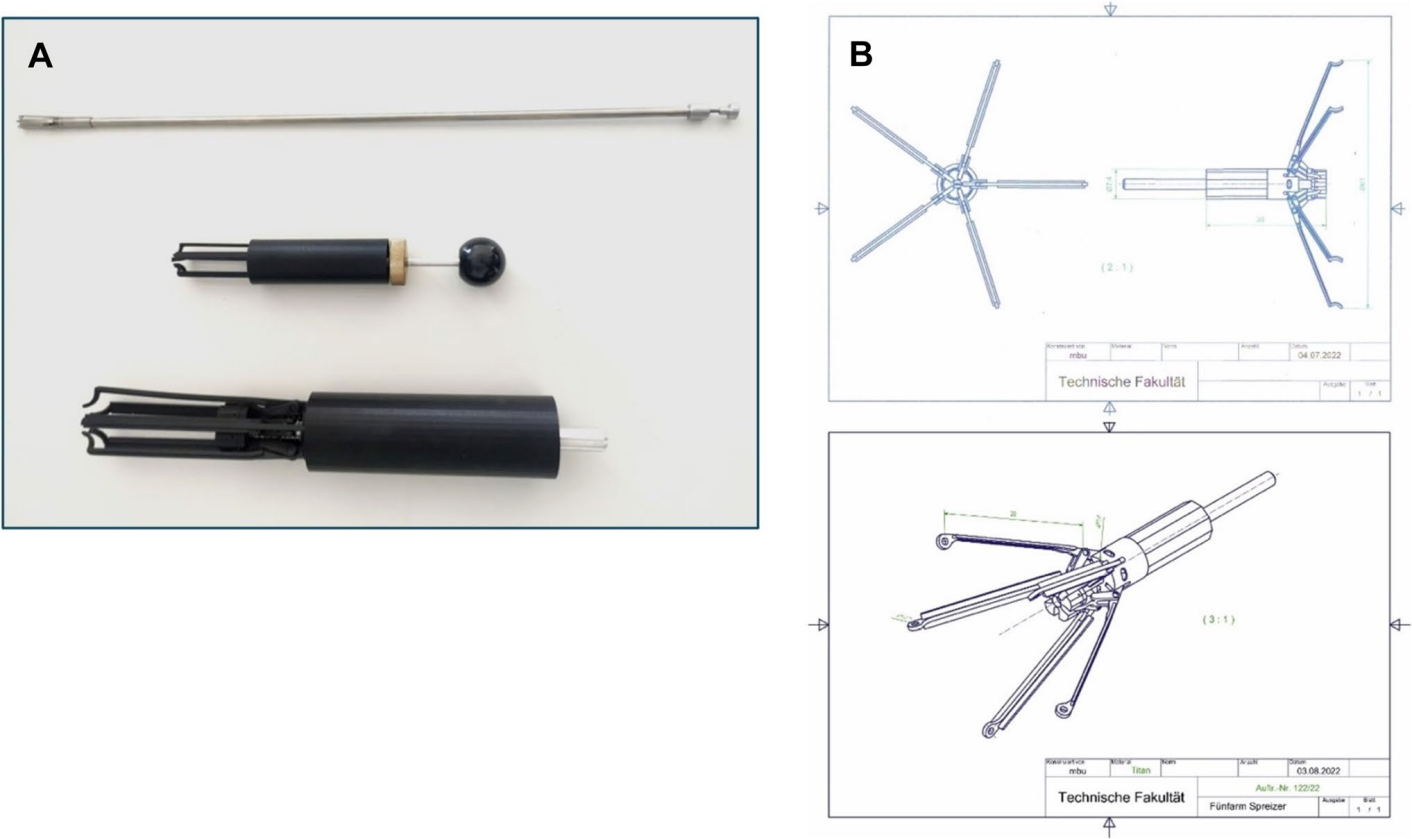
Development stages of the prototype instrument. **A** multiple generations culminating in a final diameter of 0.75 cm. **B** refinement of prototype in cooperation with the technical faculty at CAU Kiel

**Fig. 3:**
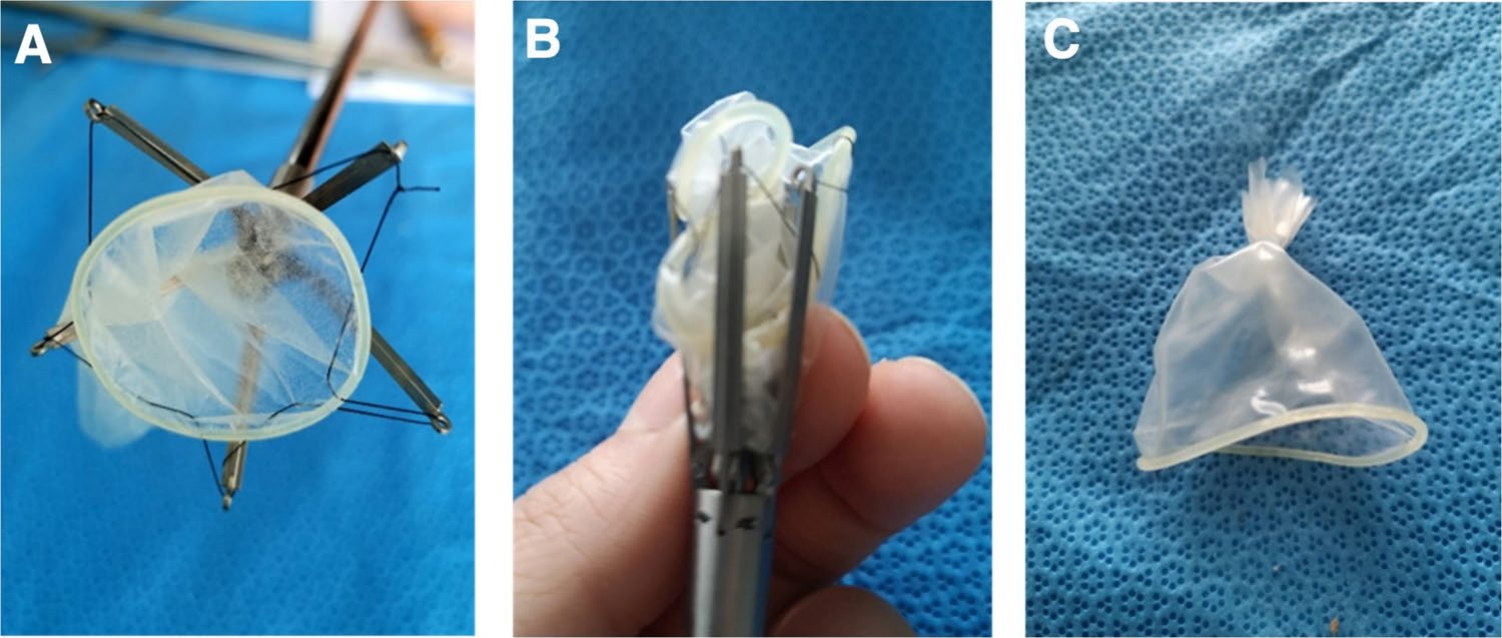
Prototype handling with customized bag. **A** expanded prototype with sewn-in bag attached via continuous suture. **B** closed prototype state, small diameter enabling insertion through standard endoscopy ports. **C** preparation of the bag

A 7.5-mm diameter titanium instrument was engineered in collaboration with biomedical engineers, drawing on insights from previous ovine fetal models (Fig. 2A, top) [6, 7]. The instrument features five retractable arms with eyelets, to which a protective bag is attached using a continuous 6-0 Prolene suture. By turning a screw mechanism, the arms expand radially to deploy the bag; upon release, they retract to clasp the bag centrally, allowing precise placement and retrieval (Fig. 3A, B).

### Bag design for gastroschisis coverage

Latex condoms (Preventivo Sensitive) were selected due to their elasticity, size, and prior use in animal models. After blotting the lubricated surface, each bag was trimmed to 4 cm in length and closed at one end with a 4-0 silk suture, facilitating handling during placement (Fig. 3C).

### Simulated fetus with gastroschisis

**Fig. 4:**
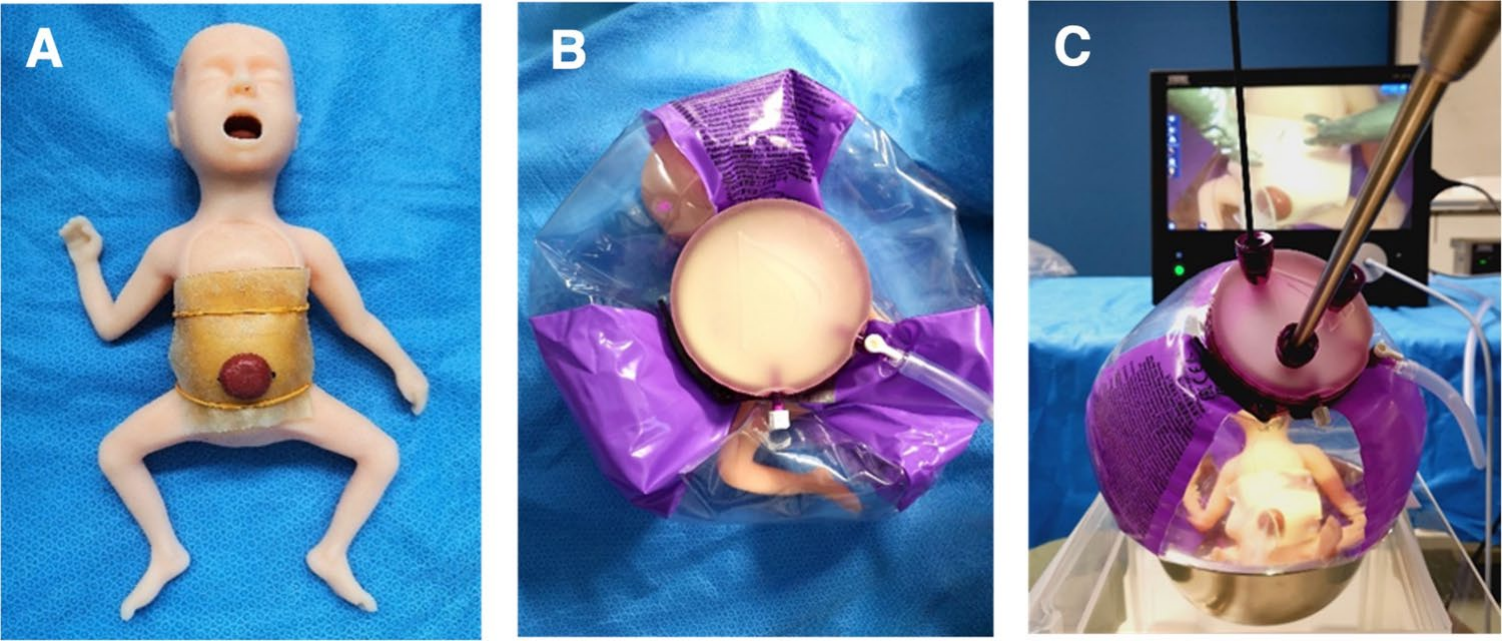
Fetal Simulator and low-fidelity uterus model. **A** Micro-Preemie Simulator with gastroschisis and latex abdominal wall, realistic measurements (weight: 0.91 kg, length: 30 cm, head circumference: 23 cm, abdomen circumference: 22 cm). Latex membranes attached using rubber bands and pins. **B** uterus model without and **C** with CO_2_ insufflation

A commercially available premature infant simulator (Nasco Healthcare, Life/form® Micro-Preemie) representing a 25-week fetus was used. A central abdominal incision simulated the gastroschisis defect, secured with pins. Latex membranes (DIN A7 size, 74 mm × 105 mm) mimicking the fetal abdominal wall were fabricated from air-dried liquid latex and replaced between procedures (Fig. 4A, B).

### Simulated uterus with PACI

**Fig. 5:**
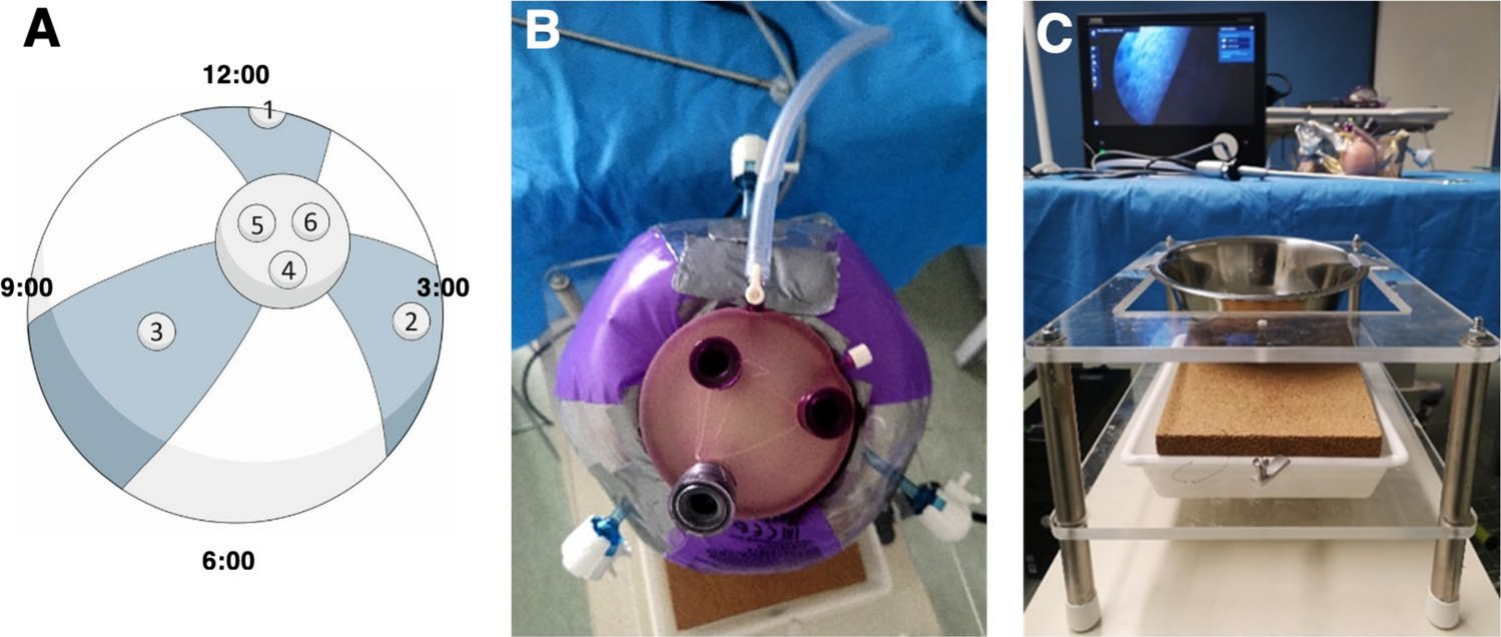
Port placement and uterus model setup. **A** sketch indicating ports: prototype (port 1), needle holders (ports 2, 3), and endoscope (port 4). **B** uterus model secured by metal bowl and framework. **C** customized endoscopy training framework elevated for ergonomic use

A transparent waterball (Bestway®) served as the uterine model. The fetal model was placed inside the waterball via an Alexis® Wound Protector-Retractor (Applied Medical, GelPoint® Advanced Access Platform) with the fetal head oriented at 6:00 o’clock. The opening at the top of the balloon was reinforced with fabric tape to prevent tearing, and the GelSeal® Cap was mounted to enable insertion of laparoscopic instruments (Fig. 4B, C). The entire assembly was placed in a metal mixing bowl secured to a custom-built endoscopy trainer frame using double-sided adhesive tape (Fig. 5C).

### Port placement for fetoscopic procedure

Three laparoscopic ports were introduced through the GelSeal® Cap (one endoscope and two needle drivers), while three additional ports were inserted directly into the balloon for optimal triangulation. A high-definition camera system (Karl Storz Telepack+) was used to document the procedures (Fig. 5B).

### Participants and experimental procedure

Eleven volunteers participated, representing varying levels of surgical experience. Participants were categorized as follows: (1) naïve: < 10 laparoscopic procedures or < 1 year of training (*n* = 4); (2) limited experience: 10–100 procedures or 1–5 years of training (*n* = 3); and (3) experienced laparoscopists: > 100 procedures or > 5 years of training (*n* = 4). Every participant performed two fetoscopic suture fixations: each with and without the prototype, in a fixed sequence. Standard laparoscopic needle drivers (Karl Storz 26173KPL and 26173KC) were inserted through any of the six available ports. A second person assisted with the prototype or fetoscope. Four square knots were placed at the 3, 6, 9, and 12 o’clock positions. Participants chose 4-0 or 5-0 silk thread (10 cm in length) and were free to select port combinations and hand dominance. After each successful knot, threads were cut to 1 cm and removed. Failed knots were repeated until completion.

### Subjective assessment

**Fig. 6:**
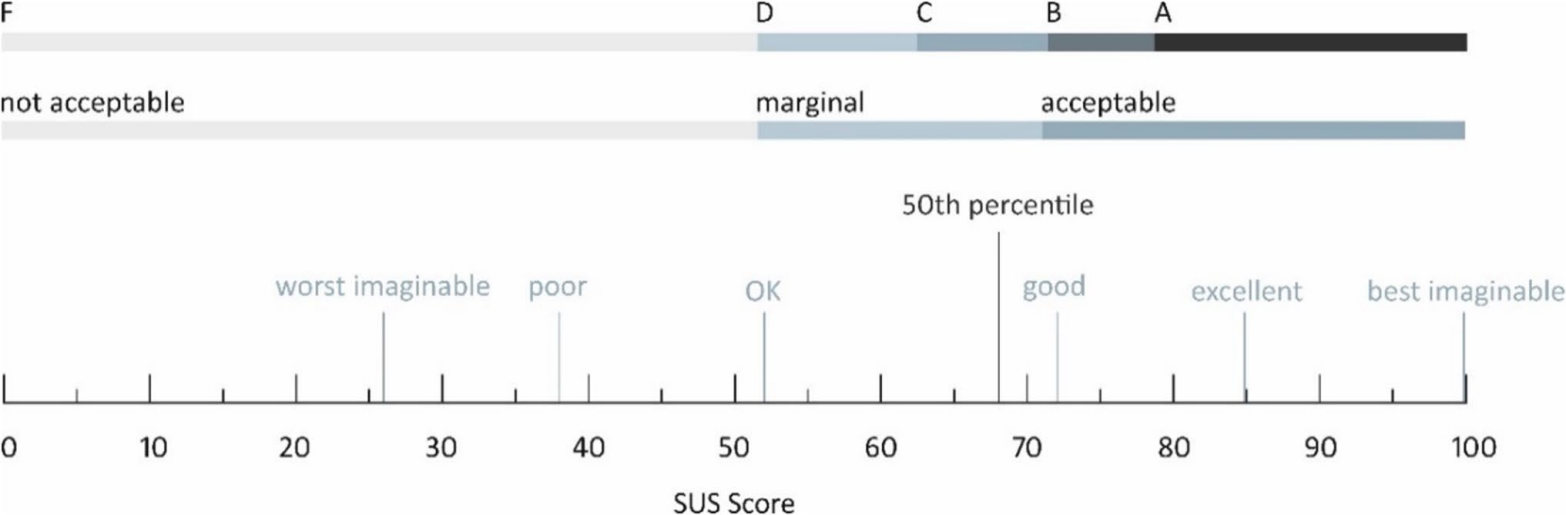
SUS total score interpretation scheme: adjective rating, acceptability, and school grading scales according to Bangor et al. [11]. Scores above 68 are above average; scores below 68 are below average [12]

After each knot row, participants completed a structured two-page questionnaire. Ten Likert-scale items assessed difficulty and handling (Table 1). Two open-ended questions addressed perceived challenges and successes. After using the prototype, the system usability scale (SUS) by John Brook was also completed [10, 11]. SUS items were scored from 0 (strongly disagree) to 4 (strongly agree), summed, and multiplied by 2.5 to yield a final score between 0 and 100 (Fig. 6).

**Table 1:** Modification of the 10 items from the original SUS

Item		Points
1	I think that I would like to use this prototype frequently	0-1-2-3-4
2	I found the prototype unnecessarily complex	4-3-2-1-0
3	I thought the prototype was easy to use	0-1-2-3-4
4	I think that I would need the support of a technical person to be able to use this prototype	4-3-2-1-0
5	I found the various functions in this prototype were well integrated	0-1-2-3-4
6	I thought there was too much inconsistency in this prototype	4-3-2-1-0
7	I would imagine that most people would learn to use this prototype very quickly	0-1-2-3-4
8	I found the prototype very cumbersome to use	4-3-2-1-0
9	I felt very confident using the prototype	0-1-2-3-4
10	I needed to learn a lot of things before I could get going with this prototype	4-3-2-1-0

The points to be awarded for the five response options on the Likert scale from “strongly disagree” to “strongly agree” are also listed. Note the reverse scoring for negatively formulated statements

### Objective data collection

The primary objective measure was knotting time (needle entry to knot completion). All procedures were recorded and cataloged. Data were logged in a comprehensive Excel file including knot number, participant ID, assistance, handedness, port configuration, use of prototype, fetal orientation, and thread type.

### Statistical analysis

Descriptive statistics (median, interquartile range) were used for non-parametric data. Wilcoxon rank-sum tests assessed paired comparisons. Mann–Whitney *U* tests were applied for unpaired data. Significance was set at *p* < 0.05 (two-tailed). Statistical analysis was performed using SPSS (IBM, v29; 13th September 2022 for Microsoft Windows) and JASP (v0.95.0; 26th July 2025 for Apple macOS).

## Results

Prior to the commencement of experiments, series of knot placements were conducted for trial purposes, providing grounds for adjustments to the experimental setup, including the avital fetus and uterus models.

### The fetal model

The commercially available fetal model performed well and required minimal modification. The smaller version of the gastroschisis attachment was chosen because the larger version detached during multiple attempts to secure it, despite using various adhesives and needles. The larger model’s own weight caused it to fall off the docking site with minimal manipulation.

### The inanimate uterus model

Several sizes and materials of balls were tested for the uterine model. While some were too small to accommodate the fetal model, others lacked the necessary durability. Ultimately, laparoscopic standard instruments were introduced through the clamped GelSeal® Cap using three low-profile ports. However, these ports had an insufficient diameter for smooth instrument insertion, so Ports 1 and 4 were replaced with larger models. The new ports provided improved flexibility and better articulation. Carbon dioxide insufflation was well managed during all trials, never exceeding 8–10 mmHg. After the tenth participant, the model had to be recreated due to significant air leakage from wear, but no material differences were found that affected the overall comparability.

### Prototype development

The instrument prototype underwent iterative development. Initially, a virtual 3D model was created based on feedback from pediatric surgery specialists. This was followed by physical fabrication at progressively smaller scales (1:7, 1:3, and full scale). The final prototype featured five fine metallic arms, which replaced the initial wire design. These arms provided greater stability and allowed for a reduced instrument diameter. The hooks at the arm ends were replaced with loops for suturing the retrieval bag in place. This modification effectively prevented the bag from detaching or slipping during use. A continuous suture passing through the loops was adopted to secure the bag after testing different fixation methods. Initially, single interrupted sutures were used, but this approach led to either excessive tension or inadvertent damage to the bag. The continuous suture was more reliable for bag fixation.

### Suturing time and success rate

A total of 181 knots were performed, but 5 were excluded from the analysis due to breakage of the suture material or incomplete knot series. This left 176 valid knots for evaluation. The 12:00 o’clock position was excluded from the statistical analysis due to the difficulty all participants had in handling the knotting at that location, independent of their prior minimally invasive surgery (MIS) experience. This left a total of 132 knots for evaluation (Table 2). The median duration for completing a knot without the prototype was 118.5 s. With the prototype, the time increased slightly to 120.5 s (*p* = 0.98).

**Table 2:** Median knot duration for both consecutive knot rows

Knot position	*N*	Knotting time without prototype in sec			Knotting time with prototype in sec		
		25th	Median	75th	25th	Median	75th
Knot row 1							
3.00	22	90.00	120.00	171.00	118.00	134.00	197.00
6.00	22	93.00	150.00	217.00	95.00	117.00	147.00
9.00	22	114.00	138.00	178.00	114.00	148.00	325.00
*12.00*	*22*	*92.00*	*116.00*	*237.00*	*135.00*	*197.00*	*313.00*
Knot row 2							
3.00	22	98.00	118.00	176.00	77.00	121.00	145.00
6.00	22	69.00	109.00	134.00	82.00	97.00	141.00
9.00	22	71.00	103.00	114.00	86.00	119.00	156.00
*12.00*	*22*	*92.00*	*148.00*	*220.00*	*137.00*	*167.00*	*370.00*

12 o’clock position in italics since it has been excluded from the statistical analyses

### System usability score (SUS)

**Fig. 7:**
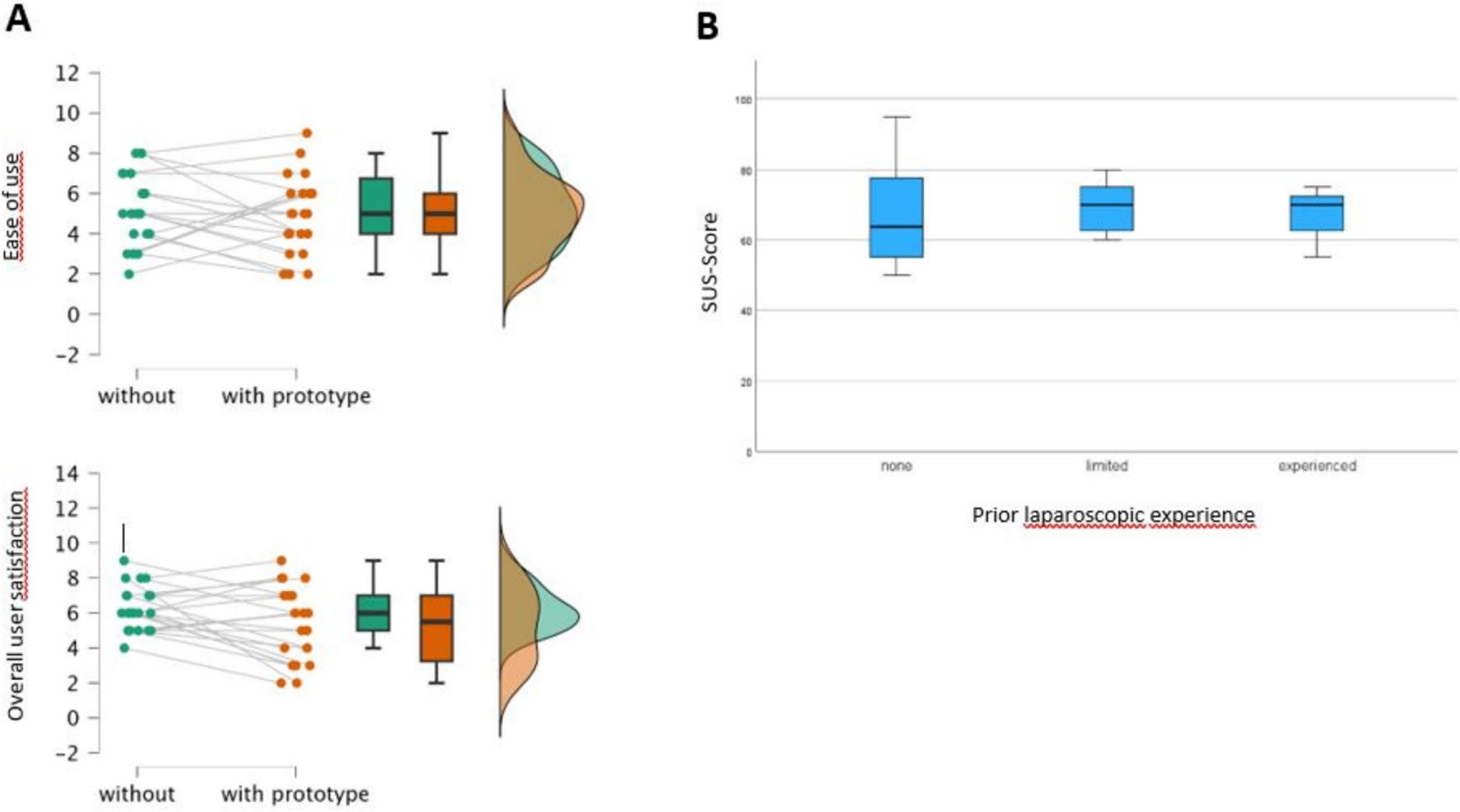
**A** Likert-scale evaluation for overall ease of use and user satisfaction with and without the prototype. **B** SUS scores by participants’ prior MIS experience

The median SUS was 70, with the 50th percentile at 68. There was no significant difference in the usability scores between participants with no prior experience in laparoscopic procedures and those with extensive MIS experience (Fig. 7B, *p* = 0.43).

### Subjective satisfaction and experienced facility

Likert-scale analysis did not result in a significant difference between using the prototype and conducting knots without it, both resulting in a median score of 5/10 (Fig. 7A, *p* = 0.67). In contrast, it showed a significant decrease in satisfaction among when using the prototype with a median of 5/10 in comparison to not using the prototype with a median of 6/10 (Fig. 7A, *p* = 0.01).

## Discussion

### The fetal model

The fetal model, designed to represent a fetus at the 25th week of gestation, offers anatomically accurate dimensions crucial for fetal surgical interventions. The reusability of the model ensures a cost-effective solution while preserving environmental resources. This commercially available model includes interchangeable attachments beyond those for gastroschisis simulation, such as those for spina bifida. Custom attachments for specific research questions can be easily created using 3D printing technology, adding flexibility for future research needs.

### The inanimate uterus model

The uterus model, with its straightforward construction, proved to be particularly cost-effective and provides an easily reproducible solution, especially valuable in educational and research settings. Its non-viable nature allows for experimental studies to be conducted without the need for animal testing in the early phases of research. Additionally, modifications, such as fluid infusion to simulate amniotic fluid instead of gas insufflation, could be incorporated depending on the specific research question and training objective.

### The prototype

The prototype demonstrated its ability to secure the bag over the gastroschisis, preventing dislocation during suturing and improving visibility. These features are particularly beneficial in fetal surgeries performed under amnioinfusion compared to traditional PACI [13]. As participants gained experience with the prototype, they adapted quickly due to the intuitive handling. The metallic arms enhanced the surgeon’s orientation, enabling quicker identification of the optimal knot placement. However, further improvement in fine mechanics, such as the addition of an articulating function, would improve the prototype’s operation and stability against vibrations.

### The participants

While expert evaluation is essential for assessing clinical efficacy or advanced procedural skills, the objective of this study is the early-stage usability validation of a novel surgical prototype. In accordance with evolving human factors practices (IEC 62366, FDA 2016 & 2022 draft; see Web reference list), early usability assessments should involve users across the expertise spectrum [14]. Studies confirm that novice and experienced users reveal complementary usability issues the former uncovering design flaws experts compensate for [15]. Moreover, integrating formative usability assessments with clinical evaluations strengthens device validation. To control for individual skill differences and learning curve effects, we adopted a paired intra-individual design, enabling each participant to serve as their own control—a methodology aligned with risk-based usability frameworks that efficiently identify critical use errors while conserving resources [16].

### The procedures

According to our results, the mean time required for patch suturing with the prototype—regardless of the previous MIS experience of the participant—was slightly longer mainly since the positioning of the rear knot (12 o’clock position) proved to be unfeasible due to the spatial constraints and limited visibility with the prototype. We took this as a reason to exclude the 12 o’clock position from statistical analysis to prevent systematic error. On the other hand, this reduced the number of knots to only 132, which may allow individual outliers to disproportionately affect the results.

### Feasibility of SUS

Since first publicized, the SUS has been widely used and validated. It is suitable for assessing usability and is an established method for quantitative analysis. By surveying multiple participants, a global overall assessment is derived from the mean of the scores of all participants. Sample size and reliability are not related, so the SUS can also be used with very small samples (8–12 participants) to make a good assessment of usability [17]. In our study, a mean SUS score of 68 indicates that the usability of the prototype is average and, according to the translation scheme (Fig. 6), can be considered acceptable, although detailed contextualization is necessary for interpretation. When comparing individual SUS scores with objectively measured suturing time and success rate of the same series of knots, a participant’s SUS score seems to correlate with their individual task performance. The SUS score is a quantitative metric and does not encompass all facets of user experience. Supplementary qualitative investigations, such as user interviews or usability tests, are necessary to identify specific challenges and make targeted improvements. Therefore, objective factors such as knotting time, success rate, type of errors, and qualitative product defects should also be considered in the overall assessment. Nonetheless, the result indicates that investing in the further development of the prototype is worthwhile.

## Limitations

### Experimental setup and identified limitations

The experimental setup revealed both strengths and weaknesses. To fully understand the potential for improvement, it is essential to identify product deficiencies and sources of error so that they can be addressed in the next iteration of the prototype. The qualitative evaluation was based not only on open-ended participant interviews but also on direct observations made by the assistant and the observer during the experiment.

### Limitations of the prototype instrument

All participants reported difficulties with the prototype, especially with bag displacement. Repeated repositioning of the bag before securing the first knot prolonged the suturing process. Additionally, careful handling was necessary to avoid bending the delicate metal arms and loosening the terminal screw. Over repeated use, the prototype’s movements became more rigid, likely due to wear and tear, particularly affecting the fine components such as the screw. Sharp edges on the prototype also increased the risk of cutting the suture or the bag.

### The 12 o’clock knot

The 12 o’clock knot position revealed a critical limitation of the current prototype design: limited mechanical articulation and visual access. Future design iterations will address these issues through enhanced flexibility, optimized tip mechanics, and an alternative fixation strategy, before proceeding to animal models.

### Limitations of the fetal model

While the fetal model provided a valuable simulation, it does not account for spontaneous fetal movements that would naturally occur in an in vivo environment. These movements, facilitated by amniotic fluid, could complicate the procedure in real life. Additionally, the latex membrane of the model showed a loss of elasticity upon exposure to sunlight, necessitating either immediate fabrication or careful storage to protect the model from heat and light damage. Regular replacement of the membrane after each use is essential to maintain the model’s integrity.

### Limitations of the uterus model

The ex vivo uterus model used in the study lacks some of the complexities of a living uterus. For instance, participants did not have to consider complications such as chorioamniotic membrane separation (CMS), bleeding, or damage to the fetus during interventions [18]. Furthermore, amniotic fluid in an in vivo setting could contribute to bag dislocation, making fixation more challenging. Therefore, alternative methods, such as underwater adhesives or other forms of bag fixation, should be explored to simplify the procedure and improve surgical outcomes [19, 20].

## Future directions

Despite these constraints, the prototype and study design exemplify a responsible and ethically sound approach to early-stage surgical innovation. The use of a reproducible, animal-free model adheres to the principles of refinement and replacement (3R), allowing repeated testing and iterative improvements before advancing to higher fidelity models. (Directives of the European Parliament and the DFG-German Research Foundation, see Web reference list).

Going forward, the next stages of development must include

- mechanical refinement of the prototype based on observed failure points,
- validation by experienced fetal surgeons in randomized crossover designs,
- preclinical testing in euthanized or anesthetized animal models (e.g., pregnant sheep),
- integration of alternative bag fixation methods suitable for amniotic fluid environments, and
- comparison to existing fetoscopic tools, where available, to assess relative efficacy and safety.

Ultimately, together with a comprehensive understanding of the pathophysiology and accurate prenatal diagnosis to ensure minimal maternal risk and fetal survival, these steps aim to make fetal surgery for gastroschisis a viable treatment for human fetuses [21].

## Conclusion

Our study demonstrates that the application of a novel instrument prototype may facilitate fetoscopic bag placement in the prenatal management of gastroschisis. Despite the benefit of secure coverage of gastroschisis, we observed relevant difficulty in bag fixation and therefore prolonged time of the procedure. This leads to the necessity for further refinement of the instrument. After optimizing the instruments and procedures in inanimate models, future experiments must be conducted in a live fetal model before commencing. Lastly research on prenatal diagnosis must result in a reliable diagnosis of the correct malformation. Only these combined efforts can result in the most accurate and timely surgical treatment as required by the complexity and severity of complicated gastroschisis.

## Web references (last accessed 07/04/2025)

https://eur-lex.europa.eu/legal-content/EN/TXT/PDF/?uri=CELEX:02010L0063-20190626

https://www.dfg.de/resource/blob/309210/f6c41e7837a3936896dad950a83c0e32/handreichung-sk-tierversuche-data.pdf

https://www.fda.gov/media/80481/download

## Abbreviations

CMS: Chorioamniotic separation
ICC: Intestinal interstitial cells, i.e., Cajal cells
MIS: Minimally invasive surgery
PACI: Partial amniotic carbon dioxide insufflation
SUS: System usability scale
VAD: Viscero-abdominal disproportion

## References

[1] Kluth D, Lambrecht W (1996) The pathogenesis of omphalocele and gastroschisis: an unsolved problem. Pediatr Surg Int 11:62–66. 10.1007/BF00183727 24057518 10.1007/BF00183727

[2] deVries PA (1980) The pathogenesis of gastroschisis and omphalocele. J Pediatr Surg 15:245–251. 10.1016/S0022-3468(80)80130-8 6445962 10.1016/s0022-3468(80)80130-8

[3] Hoyme HE, Higginbottom MC, Jones KL (1981) The vascular pathogenesis of gastroschisis: intrauterine interruption of the omphalomesenteric artery. J Pediatr 98:228–231. 10.1016/S0022-3476(81)80640-3 6450826 10.1016/s0022-3476(81)80640-3

[4] Bergholz R, Boettcher M, Reinshagen K et al (2014) Complex gastroschisis is a different entity to simple gastroschisis affecting morbidity and mortality—a systematic review and meta-analysis. J Pediatr Surg 49:1527–1532. 10.1016/j.jpedsurg.2014.08.001 25280661 10.1016/j.jpedsurg.2014.08.001

[5] Joyeux L, Belfort MA, Coppi De et al (2021) Complex gastroschisis: a new indication for fetal surgery? Ultrasound Obstet Gynecol 58:804–812. 10.1002/uog.24759 34468062 10.1002/uog.24759

[6] Krebs T, Boettcher M, Schäfer H et al (2014) Gut inflammation and expression of ICC in a fetal lamb model of fetoscopic intervention for gastroschisis. Surg Endosc 28:2437–2442. 10.1007/s00464-014-3494-x 24648107 10.1007/s00464-014-3494-x

[7] Bergholz R, Krebs T, Cremieux B et al (2021) Fetoscopic techniques for prenatal covering of gastroschisis in an ovine model are technically demanding and do not lead to permanent anchoring on the fetus until the end of gestation. Surg Endosc 35:745–753. 10.1007/s00464-020-07441-7 32072287 10.1007/s00464-020-07441-7

[8] Belfort MA, Whitehead WE, Shamshirsaz AA et al (2017) Fetoscopic open neural tube defect repair. Obstet Gynecol 129:734–743. 10.1097/AOG.0000000000001941 28277363 10.1097/AOG.0000000000001941

[9] Patel SK, Kashyrina O, Duru S, Miyabe M, Lim F-Y, Peiro JL, Stevenson CB (2021) Comparison of two- and three-dimensional endoscopic visualization for fetal myelomeningocele repair: a pilot study using a fetoscopic surgical simulator. Childs Nerv Syst 37:1613–1621. 10.1007/s00381-020-04999-4 33392653 10.1007/s00381-020-04999-4

[10] Brooke J (1995) SUS: a quick and dirty usability scale. Usability Eval Ind 189:4

[11] Bangor A, Kortum P, Miller J (2009) Determining what individual SUS scores mean: adding an adjective rating scale. J Usability Stud 4:114–123

[12] Brooke J (2013) Sus: a retrospective. J Usability Stud 8:29–40

[13] Belfort MA, Whitehead WE, Shamshirsaz AA et al (2017) Fetoscopic open neural tube defect repair: development and refinement of a two-port, carbon dioxide insufflation technique. Obstet Gynecol 129:734–743. 10.1097/AOG.0000000000001941 28277363 10.1097/AOG.0000000000001941

[14] Wiklund ME, Kendler J, Strochlic AY (2010) Usability testing of medical devices, 1st edn. CRC Press, Boca Raton

[15] Kiani P, Dolling-Boreham R, Hameed MS et al (2024) Usability, ergonomics, and educational value of a novel telestration tool for surgical coaching: usability study. JMIR Hum Factors 11:e57243. 10.2196/57243 39255487 10.2196/57243 PMC11422725

[16] Cook JA, Ramsay CR, Fayers P (2004) Statistical evaluation of learning curve effects in surgical trials. Clin Trials 1(5):421–427. 10.1191/1740774504cn042oa 16279280 10.1191/1740774504cn042oa

[17] Tullis T, Stetson JA (2006) Comparison of questionnaires for assessing website usability. In: Usability Professional Association Conference

[18] Amberg BJ, Hodges RJ, Rodgers KA et al (2021) Why do the fetal membranes rupture early after fetoscopy? A review. Fetal Diagn Ther 48:493–503. 10.1159/000517151 34404043 10.1159/000517151

[19] Chang J, Tracy TF, Carr SR et al (2006) Port insertion and removal techniques to minimize premature rupture of the membranes in endoscopic fetal surgery. J Pediatr Surg 41:905–909. 10.1016/j.jpedsurg.2006.01.006 16677880 10.1016/j.jpedsurg.2006.01.006

[20] Petersen SG, Gibbons KS, Luks FI et al (2016) The impact of entry technique and access diameter on prelabour rupture of membranes following primary fetoscopic laser treatment for Twin-twin transfusion syndrome. Fetal Diagn Ther 40:100–109. 10.1159/000441915 27073886 10.1159/000441915

[21] Harrison MR, Adzick NS (1991) The fetus as a patient surgical considerations. Ann Surg 213:279–291. 10.1097/00000658-199104000-00002 2009009 10.1097/00000658-199104000-00002 PMC1358346

